# Seasonal gut microbiota dynamics and colony performance under stationary and short-distance migratory beekeeping in *Apis mellifera ligustica*

**DOI:** 10.3389/finsc.2026.1890935

**Published:** 2026-10-08

**Authors:** Massimo Iorizzo, Gianluca Albanese, Cosimo Tedino, Mariantonietta Succi, Licia Maria Di Donato, Antonio de Nigris, Dalila Di Criscio, Sonia Ganassi, Luigi Ambrosone, Antonio De Cristofaro

**Affiliations:** 1 Department of Agricultural, Environmental and Food Sciences, University of Molise, Campobasso, Italy; 2 Department of Medicine and Health Sciences “V. Tiberio”, University of Molise, Campobasso, Italy

**Keywords:** 16S rRNA sequencing, *Apis mellifera ligustica*, honey bee gut microbiota, microbial ecology, migratory beekeeping

## Abstract

Honey bee gut microbiota play important roles in host nutrition, immune regulation, and health, and their composition can vary in response to environmental conditions, diet, seasonality, and beekeeping management. In this two-year field study, we investigated seasonal variation in the gut bacterial communities of *Apis mellifera ligustica* colonies maintained under stationary or short-distance migratory beekeeping management. Six colonies originating from the same experimental apiary were assigned to stationary or migratory management, with three migratory colonies seasonally transferred to Mount Taburno during summer and three stationary colonies remaining at Castelpoto. Bacterial communities were characterized by high-throughput sequencing of the V1–V3 region of the 16S rRNA gene. The gut microbiota was consistently dominated by characteristic honey bee-associated taxa, particularly *Gilliamella* and *Snodgrassella*, although their relative abundances varied substantially across seasons and years. Alpha-diversity analyses based on harmonized genus-level relative abundance profiles revealed significant seasonal variation in both years, whereas no significant overall effect of management system was detected. Similarly, PERMANOVA of Bray–Curtis dissimilarities revealed significant seasonal structuring of bacterial community composition in 2022 and 2023, while stationary versus migratory management did not significantly explain overall community variation. The persistence of dominant gut-associated taxa across sampling periods indicates compositional continuity of major bacterial community members despite temporal variation, but does not by itself demonstrate functional stability or microbial resilience. Colony-performance measurements showed significantly higher annual honey production in migratory than in stationary colonies in 2022, whereas no significant differences in the investigated performance parameters were detected between management systems in 2023. Thus, the productive response was not consistent between years. Although temporal variation in gut bacterial communities occurred alongside differences in colony performance, the present study does not establish a causal or functional relationship between microbiota composition and productivity. Overall, these findings identify seasonality as the predominant factor associated with variation in the gut bacterial communities of the investigated colonies and provide no evidence for a consistent overall effect of short-distance migratory management on bacterial community diversity or structure. Further studies involving larger numbers of independent colonies and apiaries, together with direct functional analyses of the microbiome and characterization of dietary and environmental factors, are required to clarify the consequences of migratory beekeeping for honey bee microbiota and colony performance.

## 1 Introduction

In recent years, honey bee (*Apis mellifera*) colonies have experienced significant population declines, creating major challenges for beekeepers and threatening agricultural ecosystems that rely on bee pollination (1, 2). Colony mortality and morbidity result from multiple interacting stressors, with Varroa mites as a major driver, while pathogens, pesticide exposure, nutritional deficiencies, and management practices further exacerbate losses (3–5).

The gut microbiota of the honey bee represents a relatively simple but highly specialized community, primarily dominated by five core bacterial lineages: *Gilliamella*, *Snodgrassella*, *Bombilactobacillus* (formerly Lactobacillus Firm-4), *Lactobacillus* (formerly Firm-5), and *Bifidobacterium* (6). In addition to these core members, other genera such as *Bartonella*, *Commensalibacter*, and *Frischella* are frequently detected in the gut but are not considered part of the ‘core’ microbiome (7, 8). Both core and non-core bacterial groups play pivotal roles in maintaining bee health by contributing to nutrient metabolism, pathogen resistance, detoxification of dietary components, and regulation of immune activity (9–11). Newly emerged workers acquire their characteristic gut microbiota primarily through social interactions within the hive, including contact with nest-mates and trophallaxis (9, 12–14). Although the honey bee gut microbiota is characterized by a relatively conserved set of core bacterial lineages, differences among individuals may occur in the relative abundance of these taxa and in the presence of transient or opportunistic bacteria (7). The gut microbiota affects both individual and colony-level health, and disturbances leading to dysbiosis can reduce host fitness and resilience (15–18). Given the central role of the gut microbiota in honey bee health, elucidating its composition and the factors driving dysbiosis is essential (19, 20). The composition of the bee gut microbiota is significantly affected by seasonal changes, primarily driven by diet, temperature, pathogenic infection, and ecosystem features (8, 14, 21–31). These changes affect the health of honey bees by strengthening or weakening their immunity, making them more or less susceptible to disease (6, 23). In a recent study, Braglia et al. (32) found that different pollens do not significantly affect the core gut microbiome composition. However, the effect of different diets on the microbiome suggests an indirect impact on the immune system response by modulating and influencing the synthesis of certain immune-related peptides (32).

Migratory beekeeping, practiced worldwide, involves relocating colonies in synchrony with floral cycles to optimize honey yields and support commercial pollination services (33). In the United States, millions of beehives are transported to provide pollination for crops such as almonds, blueberries, and cranberries (34, 35), with similar practices observed in Europe (36–38), and Asia (39–42). While providing essential pollination services, migratory beekeeping can affect biodiversity and honey bee health, potentially increasing or decreasing pollinator diversity depending on agricultural intensity and the health of native populations (43, 44). Short-distance migratory beekeeping is practiced not only in Europe but also in Australia and China at national or regional levels to exploit successive flowering periods, enabling the production of monofloral honeys and supporting colony adaptation to increasingly challenging conditions associated with hotter and drier summers (36). Some studies indicate that migratory beekeeping may negatively affect the health of honey bees (45, 46), while also increasing susceptibility to parasites and infectious diseases (36, 47–52). Cestaro et al. (53) evaluated the presence and prevalence of *Paenibacillus larvae, Nosema apis, Nosema ceranae* and *Varroa destructor* in colonies from the east-central region of São Paulo state, Brazil, in two different seasons and under two different management systems (stationary vs. migratory). The results demonstrate that seasonality affects the health of *A. mellifera* colonies, but this does not depend on the type of management adopted (stationary or migratory) (53). Recent studies have suggested that migratory beekeeping may be associated with changes in honey bee gut microbial diversity and composition (54). Such changes may have biological relevance because the gut microbiota contributes to nutrient metabolism and host defense; however, changes in microbial diversity alone should not be interpreted as evidence of impaired microbiome function or colony health (6). Advances in high-throughput sequencing and bioinformatics have greatly enhanced our understanding of the honeybee microbiome and its role in health (55). However, knowledge remains limited regarding the effects of migratory beekeeping on the gut microbiota of *A. mellifera*. A recent study by Nishanthini and Kanagarajan (54) on the Indian honeybee (*Apis cerana indica*) highlighted differences between the microbiomes of bees from different migratory sites. However, *A. cerana* (Eastern honey bee) and *A. mellifera* (Western honey bee) exhibit significant phenotypic and productive differences, largely due to their evolutionary adaptation to different environments (56, 57).

*A. mellifera ligustica* is one of the most widely kept and popular subspecies of *A. mellifera* in global beekeeping, valued for its docility, high productivity, and adaptability to diverse climates (58–60).

In this study, using Next Generation Sequencing (NGS) technology, we compared seasonal variation in the gut microbiota of honey bee colonies managed under stationary and short-distance migratory beekeeping. To our knowledge, this is the first two-year field study to investigate seasonal gut microbiota dynamics in *A. m. ligustica* under these two management conditions. We also evaluated colony performance parameters under stationary and migratory management.

## 2 Materials and methods

### 2.1 Samples collection

The study was conducted over two consecutive years (2022 and 2023) in the Campania region (southern Italy), specifically in the municipality of Castelpoto (41°08′42″ N; 14°42′14″ E; 280 m a.s.l.) and at Piano Melaino on Mount Taburno (41°06′44″ N; 14°35′12″ E; 1350 m a.s.l.), within the Taburno-Camposauro Regional Park. Six *A. m. ligustica* colonies were initially located in the same experimental apiary in Castelpoto. All colonies were headed by sister queens derived from the same genetic line and were selected to have a comparable initial level of colony development. The use of sister queens allowed partial standardization of the host genetic background across experimental groups, although sister queens are not genetically identical. The original experimental scheme confirms the common genetic lineage and comparable initial colony development.

The six hives were assigned to two management groups. In the stationary beekeeping group, three hives (nos. 3, 4, and 6) remained at the Castelpoto site throughout the experimental period. In the migratory beekeeping group, three hives (nos. 1, 2, and 5) were seasonally transferred from Castelpoto to Piano Melaino on Mount Taburno, where they remained during the summer period and had returned to Castelpoto by the autumn sampling. In 2022, the migratory colonies were transferred to Mount Taburno on 25 June, while hives 3, 4, and 6 remained at Castelpoto. The same experimental management scheme was repeated in 2023. Microbiota data from the three stationary colonies (hives 3, 4, and 6) were previously included in a longitudinal study focused specifically on seasonal gut microbiota dynamics within the single Castelpoto apiary (61). In the present study, these data were incorporated into a broader experimental design together with three short-distance migratory colonies (hives 1, 2, and 5), with the specific aim of comparing seasonal gut microbiota dynamics between stationary and migratory management systems. The present study additionally evaluates colony-performance parameters in relation to the two management conditions.

The two sites were separated by an approximate horizontal geodesic distance of 10.5 km and differed markedly in altitude and surrounding floral resources. During summer, the Castelpoto site was mainly characterized by plants belonging to the genera *Helminthotheca*, *Trifolium*, and *Beta*. In contrast, the higher-altitude Taburno site showed a greater abundance of pollen-producing plants belonging to the genera *Echium*, *Trifolium*, *Castanea*, and *Rubus* (62, 63).

All honey bee colonies received standardized nutritional supplementation to ensure consistent dietary conditions. During winter, each colony received a single dose of 2.5 kg candied paste composed of sucrose, glucose, and fructose (Fondabee, Beernem, Belgium). At the beginning of spring, colonies were additionally supplemented with 50% (*w/v*) sucrose syrup, administered weekly for four consecutive weeks.

Microbiological sampling was performed weekly during each season in both 2022 and 2023. At each weekly sampling, fifteen returning adult forager bees were collected at the hive entrance from each colony. Individual worker age within the forager category was not experimentally standardized. After collection, the bees were immediately transported to the reference laboratory under controlled temperature conditions using liquid nitrogen and stored at −80 °C until processing. Bees collected weekly from the same colony during the corresponding season were subsequently combined to generate a single Seasonal Sampling Unit (SSU). For each SSU, all bees were processed and dissected on the same day according to Iorizzo et al. (64). For each bee, the hindgut, comprising the ileum and rectum, was aseptically dissected. The hindguts belonging to bees from the same SSU were pooled in a sterile 1.5-mL centrifuge tube. The resulting SSUs were stored at −80 °C until shipment to the DNA sequencing service provider.

Four SSUs, corresponding to spring, summer, autumn, and winter, were generated for each colony in each year. Thus, 24 SSUs were obtained annually (3 colonies × 2 management systems × 4 seasons), resulting in a total of 48 SSUs over the two-year study period. For each combination of management system, season, and year, the biological replication therefore consisted of three independent colonies (n = 3).

### 2.2 DNA extraction, amplicon library preparation and sequencing

Total genomic DNA was extracted from each Seasonal Sampling Unit (SSU) using the Stool DNA Isolation Kit (Norgen Biotek Corp., Thorold, ON, Canada) according to the manufacturer’s instructions. The bacterial community was characterized by amplification of the V1–V3 hypervariable regions of the 16S rRNA gene using the primer pair 27F (5′-AGAGTTTGATCMTGGCTCAG-3′) and 534R (5′-ATTACCGCGGCTGCTGG-3′) (65, 66). PCR amplicons were purified, quantified, and normalized before library preparation. Equimolar amounts of purified amplicons were pooled to ensure comparable sequencing depth among samples. Sequencing was conducted by Eurofins Genomics Europe Sequencing GmbH (Ebersberg, Germany) on an Illumina MiSeq platform using a paired-end 2 × 300 bp sequencing strategy.

### 2.3 Bioinformatic analysis

Raw paired-end sequences passing the Illumina chastity filter were demultiplexed according to sample-specific indices. Primer sequences were trimmed with no mismatches allowed, and read pairs lacking primer identification were discarded. Forward and reverse reads were merged when sufficient overlap was present; when merging was not possible, only high-quality forward reads were retained. Additional quality filtering removed sequences outside the expected length range and sequences containing ambiguous bases prior to downstream microbiome profiling.

Bioinformatic processing of the quality-filtered sequences was performed using the QIIME pipeline (67), as implemented within the Eurofins analytical workflow. Chimeric sequences were identified and removed using the *de novo* UCHIME algorithm (68) implemented in VSEARCH (69).

The remaining high-quality sequences were partitioned into operational taxonomic units (OTUs) using Minimum Entropy Decomposition (MED) (70). Taxonomic assignment of OTU representative sequences was performed using DC-MEGABLAST against the NCBI nucleotide (nt) reference database (release 2020-02-03). Reference sequences were considered when they showed at least 70% sequence identity across at least 80% of the OTU representative sequence. The final taxonomic assignment corresponded to the lowest common taxonomic unit shared by the best-matching reference sequences. Bacterial taxonomic abundances were normalized using lineage-specific 16S rRNA gene copy numbers following the approach described by Angly et al. (71). The resulting OTU abundance data were further processed to compute taxonomic relative abundances (%) for each sample. Subsequent data handling and numerical computations were performed using an in-house MATLAB script (version 23.2.0.2485118, R2023b). Graphical and statistical analyses were performed using Python (version 3.11.13) and R (version 4.5.0). To ensure comparability across all sampling seasons and study years, the primary diversity analyses were performed using taxonomic relative abundance profiles harmonized at the genus level (**Supplementary Dataset S1**). The 0.5% relative-abundance threshold was applied only for graphical presentation of the taxonomic profiles and was not used to filter the abundance tables employed for alpha- or beta-diversity analyses. Diversity analyses were performed using the complete harmonized genus-level relative abundance profiles. This approach was adopted because the 2022 samples were distributed across independently processed OTU datasets, precluding direct combination of the corresponding OTU feature spaces without introducing potential batch-related bias. Alpha diversity was evaluated using the Shannon and Inverse Simpson indices.

Beta diversity was assessed using Bray–Curtis dissimilarities calculated from genus-level relative abundance profiles. Principal Coordinates Analysis (PCoA) was used to visualize differences in bacterial community composition among samples. PERMANOVA was performed separately for each study year to evaluate the effects of season and management system on bacterial community composition. Because the same colonies were sampled repeatedly across seasons, the significance of the seasonal effect was assessed using 9,999 permutations restricted within colony. The effect of management system, which represented a between-colony factor, was evaluated using exact permutations of management assignments at the whole-colony level (20 possible allocations of three colonies to each management group), thereby preserving the repeated-measures structure of the experimental design. PERMDISP was applied to the same Bray–Curtis distance matrices to test for differences in multivariate dispersion among seasons and between management systems.

For 2023, for which all four seasonal sampling periods were represented within a single OTU dataset, alpha- and beta-diversity analyses were additionally performed directly from the OTU abundance table as a confirmatory analysis. OTU-level alpha diversity was evaluated using the Shannon and Inverse Simpson indices and observed OTU richness, whereas beta diversity was assessed using Bray–Curtis dissimilarities followed by PCoA, PERMANOVA, and PERMDISP using the same experimental factors and permutation structure described above. Statistical significance was set at *p* < 0.05.

### 2.4 Colony performance parameters

To assess colony performance and development, several parameters were evaluated, including colony population size, quantities of hive-stored honey and pollen, and annual honey production (72, 73). Monitoring was conducted between June and October.

#### 2.4.1 Colony population

Colony population was estimated using the Liebefeld method, based on a visual assessment of the number of adult worker bees covering both sides of each comb frame (74–76).

#### 2.4.2 Hive stored honey and pollen

Colonies were housed in Dadant–Blatt hives (47 × 30 cm frames). Each frame was virtually divided into six equal sections per side, resulting in 12 sections per frame. The proportion of each section occupied by sealed (operculated) honey and pollen was visually estimated. Assuming a concentration of 1 g/cm² of stored material, this corresponded to an average of 0.18 kg per one-sixth of a frame, as previously reported (72, 73, 77).

#### 2.4.3 Honey production

Honey productivity was determined by measuring the difference in weight between supers before and after honey extraction, following the standardized COLOSS protocol (74).

#### 2.4.4 Statistical analysis

All statistical analyses of colony-performance parameters were conducted using R (version 4.5.0). The colony was considered the independent experimental unit, with three independent colonies per management system (*n* = 3). For hive-stored honey, hive-stored pollen, and colony population, the five repeated measurements obtained during each year were first averaged within each colony to obtain a single annual value per colony. Total honey production was determined directly at the colony level as annual production. Stationary and migratory colonies were compared separately for each year using Welch’s two-sample *t*-test, which does not assume equal variances between groups. Data are reported as mean ± standard deviation (SD) of the three independent colonies. Statistical significance was set at *p* < 0.05.

## 3 Results and discussion

### 3.1 Seasonal dynamics of the honey bee gut microbiota under stationary and migratory beekeeping

The present study provides a comprehensive characterization of the seasonal dynamics of the gut microbiota associated with *A. m. ligustica* under two different beekeeping management systems, namely stationary and short-distance migratory beekeeping. The migratory colonies were transferred from Castelpoto to Mount Taburno during summer and returned to Castelpoto before the autumn sampling period, whereas the stationary colonies remained in Castelpoto throughout the study.

High-throughput sequencing of the V1–V3 region of the bacterial 16S rRNA gene revealed a taxonomically diverse bacterial community comprising 25 phyla, 43 classes, 91 orders, 159 families, and 242 genera. Taxonomic assignments reaching the species level were obtained for 72 bacterial taxa; however, given the intrinsic limitations of partial 16S rRNA gene sequencing for species-level discrimination, these assignments should be interpreted cautiously and are reported primarily as supplementary descriptive information.

### 3.2 Seasonal taxonomic restructuring of the honey bee gut microbiota

At the genus level, the honey bee gut bacterial community was consistently dominated by *Gilliamella* and *Snodgrassella*, although their relative abundances varied across seasons, years, and management groups (**Figures 1**, **2**). *Gilliamella* showed a marked increase during autumn, reaching 70.1% in stationary colonies and 66.3% in migratory colonies in 2023. Conversely, *Snodgrassella* was generally more abundant during spring and winter, reaching 52.7% in migratory colonies in spring 2022 and 51.3% in stationary colonies in spring 2023. Overall, these two dominant genera displayed contrasting seasonal patterns, with *Gilliamella* becoming more prominent in autumn and *Snodgrassella* contributing more strongly to the community during spring and winter.

**Figure 1:**
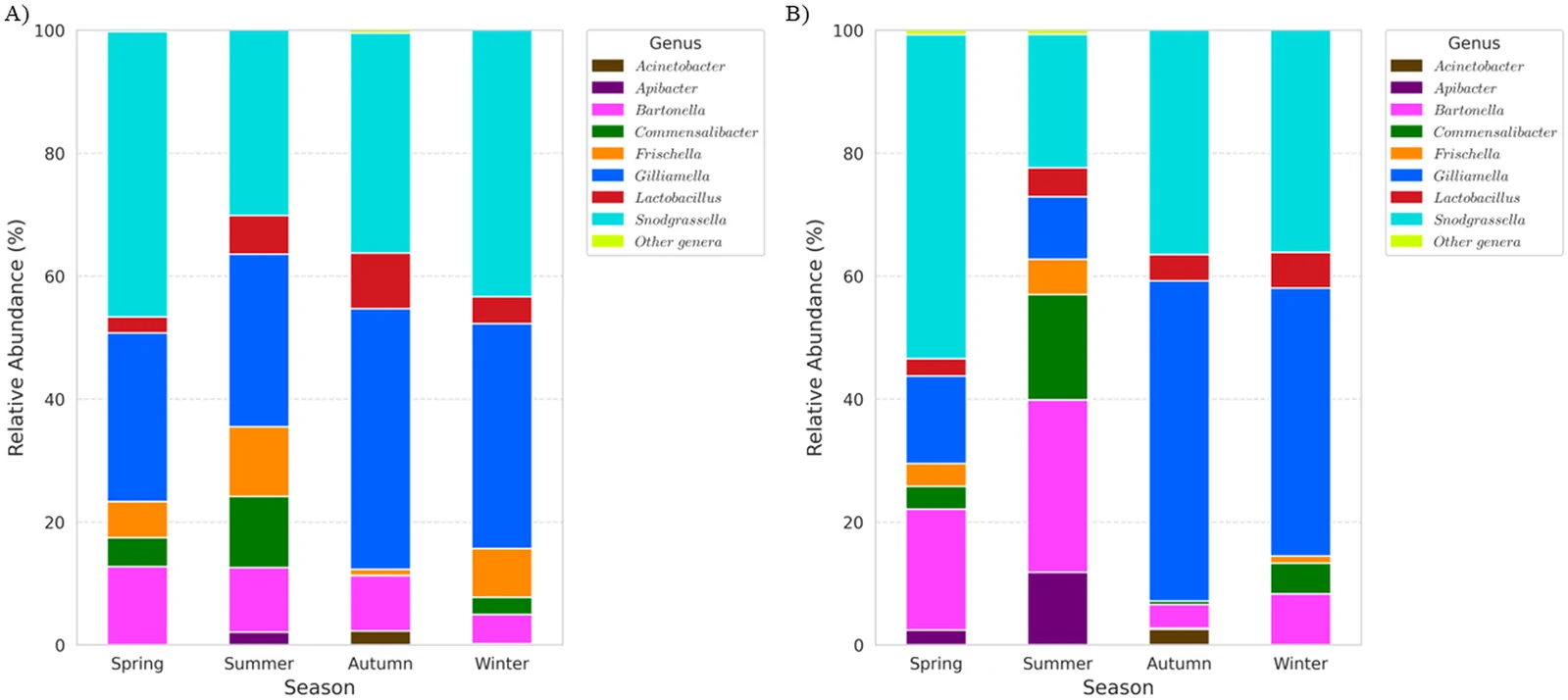
Genus-level composition of the bacterial communities associated with honey bees in 2022. Stacked bars represent the mean relative abundance (%) of the principal bacterial genera across the three independent colonies (*n* = 3) for each season under stationary **(A)** and migratory **(B)** beekeeping management. Genera not individually displayed were grouped as “Other genera”.

**Figure 2:**
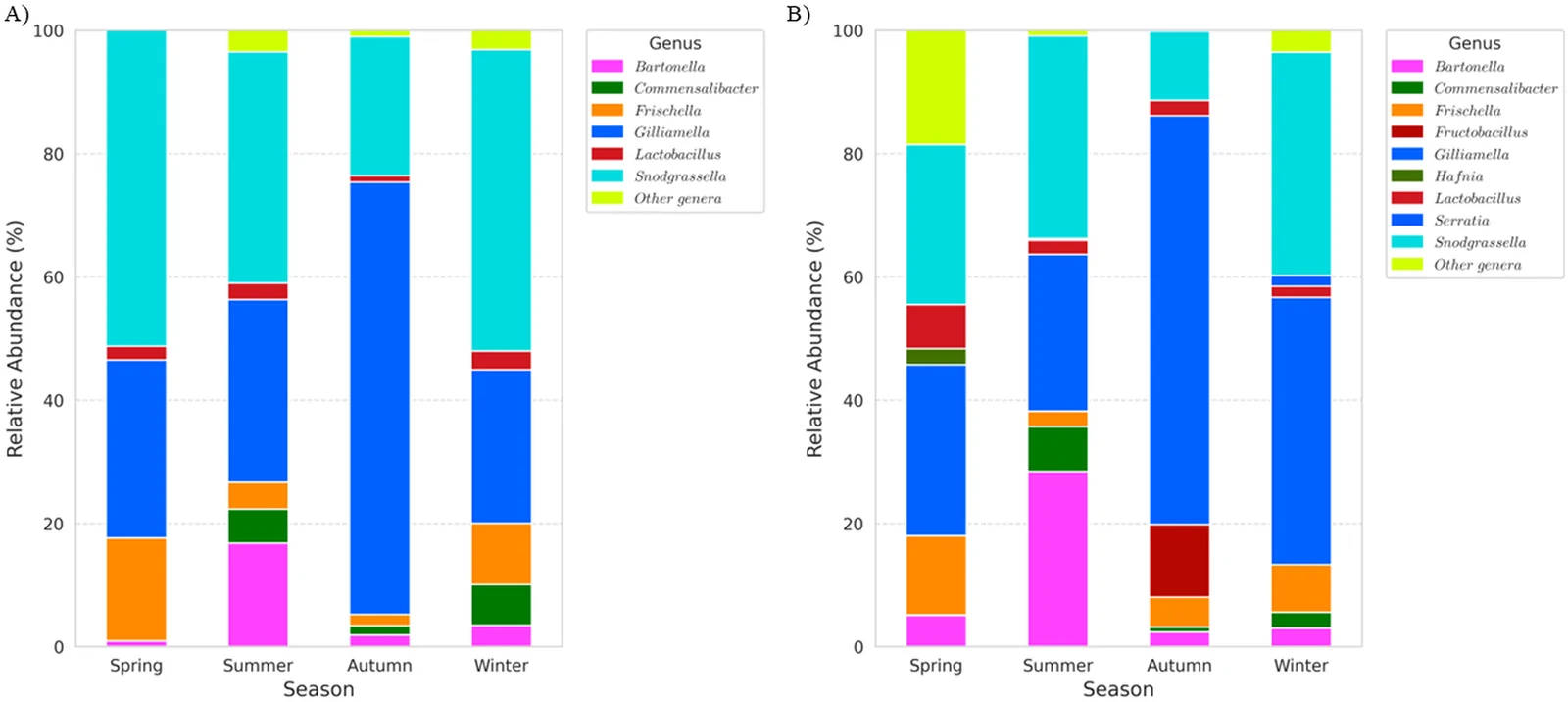
Genus-level composition of the bacterial communities associated with honey bees in 2023. Stacked bars represent the mean relative abundance (%) of the principal bacterial genera across the three independent colonies (*n* = 3) for each season under stationary **(A)** and migratory **(B)** beekeeping management. Genera not individually displayed were grouped as “Other genera”.

Among the less abundant genera, *Bartonella* showed a pronounced seasonal increase, reaching 28.0% in migratory colonies during summer 2022. *Fructobacillus* also displayed marked seasonal variation, with a pronounced increase in migratory colonies during autumn 2023. *Frischella* was detected at lower relative abundances and showed variation across seasons and years.

Overall, the genus-level profiles revealed substantial seasonal variation in bacterial community composition in both stationary and migratory colonies, with differences in the magnitude of these changes between management groups and years. Additional taxonomic profiles at the class, order, family, and species levels are provided in the **Supplementary Materials** (**Supplementary Figures S1–S4**, respectively), showing the corresponding seasonal patterns under stationary and migratory management in both study years.

### 3.3 Temporal variation in bacterial community composition under stationary and migratory management

Descriptive taxonomic profiles showed temporal variation in bacterial community composition in both stationary and migratory colonies. Differences in the relative abundance of several bacterial genera between the two management groups were observed during individual seasons, particularly during summer and autumn. However, these descriptive differences should not be interpreted as evidence of a consistent overall effect of migratory management on bacterial community structure, as the multivariate analyses did not detect a significant management effect in either study year.

The seasonal relocation of migratory colonies to Mount Taburno exposed them to environmental and floral conditions different from those experienced by stationary colonies at Castelpoto, which may have contributed to some of the taxonomic differences observed during individual sampling periods. Nevertheless, the relative contribution of floral resources, environmental exposure, and other site-associated factors cannot be disentangled from the present experimental design. Therefore, the observed taxonomic variation is more appropriately interpreted within the broader context of temporal and environmental variation rather than as evidence of enhanced microbial turnover or microbiota plasticity associated with migratory management.

### 3.4 Persistence and temporal variation of core gut symbionts

Similar temporal variation in honey bee-associated bacterial communities has been reported in relation to environmental heterogeneity, seasonal changes, and colony translocation (14, 24). In addition, the seasonal microbiota dynamics of the three stationary colonies included in the present study (hives 3, 4, and 6) were previously examined in a longitudinal analysis restricted to the Castelpoto apiary (61). That study documented seasonal variation in the relative abundance of dominant gut-associated taxa, particularly *Gilliamella* and *Snodgrassella*. In the present study, data from these stationary colonies were incorporated into a broader comparative design that also included three short-distance migratory colonies, allowing seasonal microbiota patterns to be evaluated under the two management conditions. Despite substantial temporal variation in relative abundance, dominant honey bee-associated bacterial taxa remained detectable across seasons and years. In particular, *Gilliamella* and *Snodgrassella* persisted as major members of the gut bacterial community in both stationary and migratory colonies.

Previous studies have associated these core gut symbionts with important functions related to digestion, nutrient metabolism, detoxification of dietary compounds, immune regulation, and protection against pathogens (20, 78, 79). However, microbial functions were not directly measured in the present study. Consequently, the persistence of *Gilliamella* and *Snodgrassella* should not be interpreted as evidence that their metabolic or host-associated functions remained unchanged across seasons or management conditions.

Interestingly, *Bifidobacterium*, commonly recognized as an important member of the honey bee gut microbiota, was detected at very low relative abundance in the present dataset. Previous studies have identified *Bifidobacterium* as an important symbiotic taxon, particularly associated with the rectum and hindgut compartments of honey bees (7, 79). Its low relative abundance in the present study may reflect several non-mutually exclusive factors, including ecological variation, differences in floral resource availability, gut sampling procedures, and methodological factors associated with 16S rRNA gene amplification and taxonomic profiling. More generally, amplification and taxonomic profiling based on partial 16S rRNA gene sequences may introduce taxon-dependent methodological biases. Therefore, the low relative abundance of *Bifidobacterium* should be interpreted cautiously and should not be considered evidence of its ecological absence from the investigated colonies. Overall, the persistence of dominant gut-associated taxa alongside substantial temporal changes in their relative abundances indicates that bacterial community variation occurred without the disappearance of its principal members. Nevertheless, taxonomic persistence alone cannot establish microbiome functional stability, resilience, functional redundancy, or the presence or absence of dysbiosis. Direct functional analyses would be required to determine whether the observed compositional changes were accompanied by changes in microbial activity or host-associated functions.

### 3.5 Seasonal patterns in alpha diversity

Alpha diversity was primarily evaluated using the Shannon and Inverse Simpson indices calculated from genus-level relative abundance profiles (**Figure 3**); individual diversity values and the corresponding statistical outputs are provided in **Supplementary Dataset S2**. Both indices revealed pronounced temporal variation in bacterial diversity, whereas no significant overall effect of management system was detected. In 2022, season significantly affected both Shannon diversity (*p* = 0.0001) and Inverse Simpson diversity (*p* = 0.0002), while differences between stationary and migratory colonies were not significant (*p* = 0.600 and *p* = 1.000, respectively). A comparable pattern was observed in 2023, when significant seasonal effects were detected for Shannon (*p* = 0.0019) and Inverse Simpson (*p* = 0.0014), whereas management-related differences remained non-significant (*p* = 0.700 for both indices).

**Figure 3:**
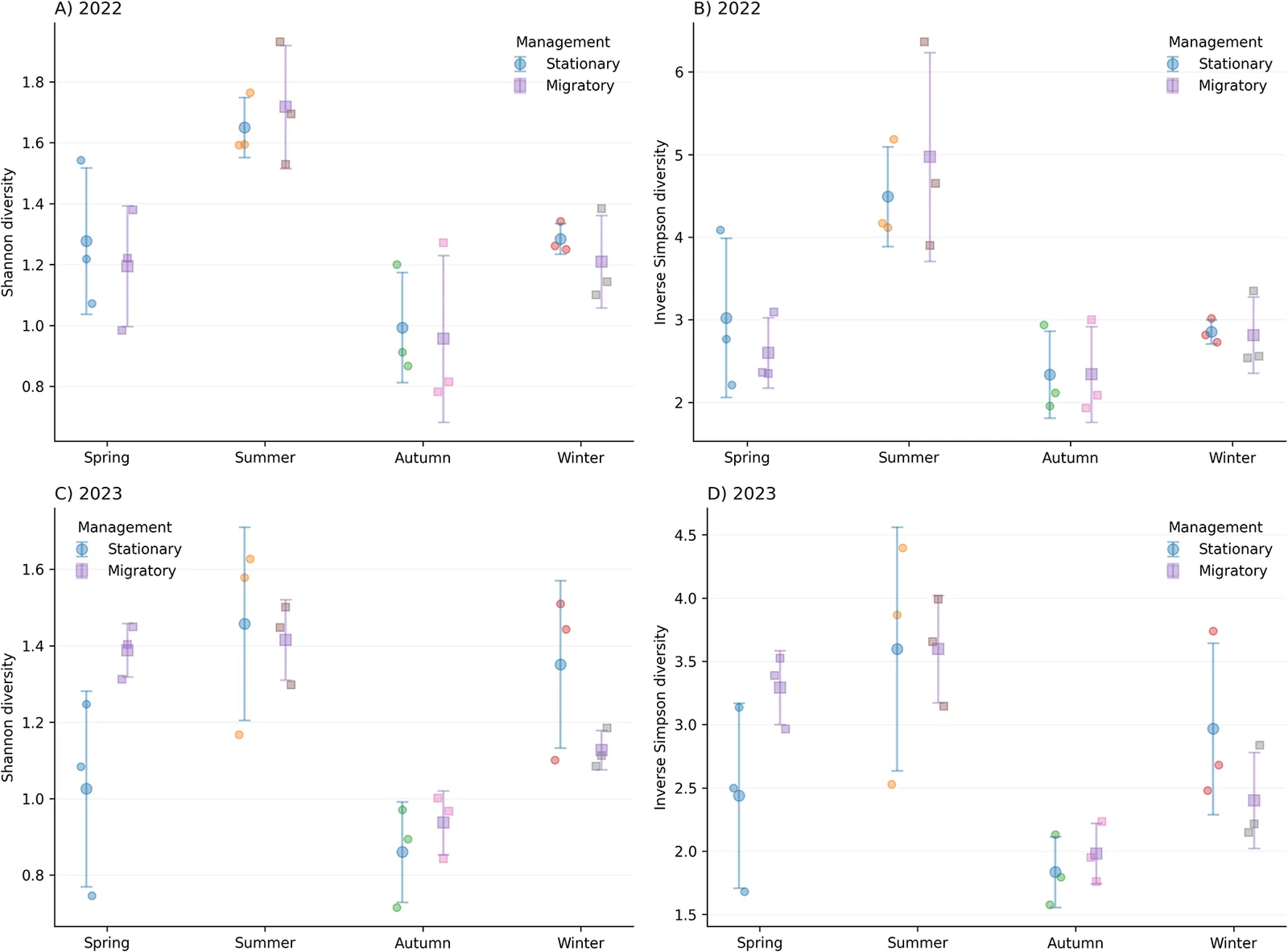
Seasonal variation in alpha diversity of honey bee gut bacterial communities under stationary and migratory beekeeping management. Shannon diversity **(A)** and Inverse Simpson diversity **(B)** are shown for 2022, whereas Shannon diversity **(C)** and Inverse Simpson diversity **(D)** are shown for 2023. Individual points represent independent colonies (*n* = 3 per management system at each seasonal sampling period), while larger symbols and error bars indicate group means ± SD. Alpha-diversity indices were calculated from genus-level relative abundance profiles.

The marked seasonal variation observed in alpha diversity is consistent with previous evidence indicating that honey bee-associated microbial communities can vary in relation to seasonal changes in diet, floral resources, colony physiology, and environmental exposure (80). Nutritional conditions, particularly the diversity and quality of pollen resources, are known to influence honey bee physiology and colony-level responses (81), providing a plausible ecological context for the temporal patterns observed in the present study. However, because diet composition was not directly linked to microbiota variation and microbial functions were not experimentally assessed, the mechanisms underlying these seasonal changes cannot be established from the present data.

Importantly, the absence of a significant overall management effect indicates that short-distance migratory beekeeping was not associated with a consistent increase or decrease in alpha diversity across the two study years. Although individual seasons and colonies exhibited differences in Shannon and Inverse Simpson values, these variations should therefore be interpreted primarily as temporal patterns rather than as evidence of enhanced microbial diversity in migratory colonies. Moreover, alpha diversity alone cannot be used to infer microbiome functional stability, resilience, functional redundancy, or the presence or absence of dysbiosis. Such properties depend on community composition and function and cannot be established solely from taxonomic diversity indices (82, 83).

As a confirmatory analysis, alpha diversity was additionally evaluated directly from the OTU abundance table for the complete 2023 dataset (**Supplementary Dataset S3**). OTU-level analyses confirmed a significant seasonal effect for both Shannon (*p* = 0.0113) and Inverse Simpson (*p* = 0.0415), whereas no significant overall management effect was detected (*p* = 0.100 and *p* = 0.200, respectively). Observed OTU richness did not differ significantly according to management system (*p* = 0.800) and showed a non-significant seasonal pattern (*p* = 0.0597). Thus, the OTU-level analysis independently supported the principal finding obtained from the harmonized genus-level profiles: temporal variation in bacterial diversity was primarily associated with season rather than with stationary versus migratory management.

### 3.6 Seasonal structuring of bacterial communities revealed by beta diversity

Beta diversity was assessed using Bray–Curtis dissimilarities calculated from genus-level relative abundance profiles. Principal Coordinates Analysis (PCoA) was used to visualize differences in bacterial community composition among samples across seasons (**Figure 4**). PCoA coordinates and the complete PERMANOVA and PERMDISP outputs are provided in **Supplementary Dataset S2**. The first two PCoA axes accounted for 74.75% of the represented variation in 2022 and 63.75% in 2023, indicating that a substantial proportion of the variation among samples was represented by the two-dimensional ordinations.

**Figure 4:**
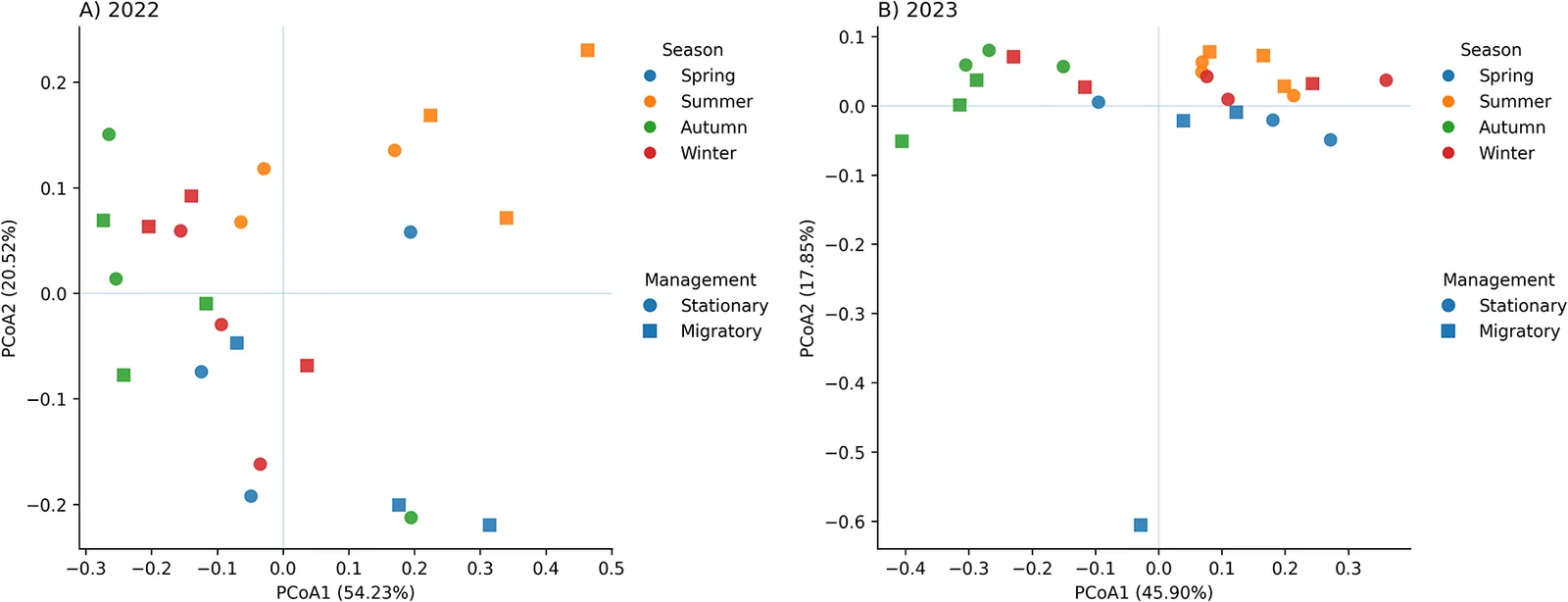
Principal Coordinates Analysis (PCoA) of honey bee gut bacterial communities based on Bray–Curtis dissimilarities calculated from genus-level relative abundance profiles in 2022 **(A)** and 2023 **(B)**. Each point represents an independent colony sampled during a given season. Colors indicate season (spring, summer, autumn, and winter), whereas symbol shapes distinguish stationary and migratory beekeeping management. The first two PCoA axes explained 74.75% and 63.75% of the total variation in 2022 and 2023, respectively.

PERMANOVA revealed a significant effect of season on bacterial community composition in both study years. In 2022, season explained 41.0% of the variation in Bray–Curtis dissimilarities (pseudo-*F* = 4.63, R² = 0.410, *p* = 0.001), whereas in 2023 it explained 46.5% of the variation (pseudo-*F* = 5.80, R² = 0.465, *p* = 0.0001). In contrast, management system did not significantly explain bacterial community variation in either 2022 (pseudo-*F* = 0.90, R² = 0.039, *p* = 0.300) or 2023 (pseudo-*F* = 0.96, R² = 0.042, *p* = 0.200). These results indicate that seasonality, rather than stationary versus short-distance migratory management, was the predominant factor associated with variation in bacterial community structure.

PERMDISP analyses were performed to determine whether the PERMANOVA results could be influenced by differences in multivariate dispersion. No significant differences in dispersion among seasons were detected in either 2022 (*p* = 0.767) or 2023 (*p* = 0.059). Similarly, multivariate dispersion did not differ significantly between stationary and migratory colonies in either 2022 (*p* = 0.100) or 2023 (*p* = 0.400). Thus, the significant seasonal effects detected by PERMANOVA were not accompanied by statistically significant differences in multivariate dispersion, although the 2023 result should be interpreted cautiously given its proximity to the conventional significance threshold.

The predominance of seasonal variation is consistent with previous evidence indicating that honey bee gut bacterial communities can vary in relation to temporal changes in diet, floral resource availability, colony physiology, and environmental exposure (80). Seasonal changes in nutritional resources may therefore provide a plausible ecological context for the temporal community patterns observed here. However, the present study did not directly test the contribution of individual dietary or environmental factors to the observed beta-diversity patterns; consequently, these potential mechanisms should be regarded as explanatory hypotheses rather than demonstrated causal relationships. As a confirmatory analysis, beta diversity was additionally evaluated directly from the OTU abundance table for the complete 2023 dataset. OTU-level PERMANOVA confirmed a significant effect of season on bacterial community composition (pseudo-*F* = 2.02, R² = 0.233, *p* = 0.0001), whereas the overall effect of management system was not significant (pseudo-*F* = 0.98, R² = 0.042, *p* = 0.400). PERMDISP detected no significant differences in multivariate dispersion among seasons (*p* = 0.1466) or between management systems (*p* = 0.100). The OTU-level analysis therefore independently supported the main pattern obtained from the harmonized genus-level profiles, showing pronounced seasonal structuring of bacterial community composition without evidence of a consistent overall differentiation between stationary and migratory management systems.

### 3.7 Colony performance under stationary and migratory beekeeping

Colony performance was evaluated over two consecutive years by measuring adult bee population, hive-stored honey, hive-stored pollen, and annual honey production. These parameters provide complementary information on colony development, nutritional reserves, and productive performance. Honey bee colony strength is closely associated with food availability, nutritional status, and environmental conditions, which can influence brood development, colony maintenance, and productivity (84). Nectar collected by foragers is converted into honey, whereas pollen represents an important nutritional resource that is stored within the hive and contributes to colony nutrition during periods of reduced resource availability (85, 86). Individual colony measurements and the corresponding sampling dates, annual colony-level values used for statistical comparisons, and complete statistical outputs are provided in **Supplementary Dataset S4**, whereas group-level annual values are summarized in **Table 1**.

**Table 1:** Hive-stored honey (kg), hive-stored pollen (kg), colony population (number of adult bees), and total honey production (kg) during 2022 and 2023 under stationary (Castelpoto) and migratory (Taburno) beekeeping practices.

Year	Management	Stored honey (kg)	Stored pollen (kg)	Colony population	Total honey production (kg)
2022	Stationary	2.03 ± 0.19	0.90 ± 0.15	19,897 ± 202	**12.20 ± 2.14^b^**
	Migratory	3.00 ± 0.68	0.90 ± 0.12	20,532 ± 1,093	**20.83 ± 3.04^a^**
2023	Stationary	3.20 ± 0.68	1.00 ± 0.13	19,150 ± 1,489	14.47 ± 5.04
	Migratory	3.19 ± 0.41	1.34 ± 0.27	20,700 ± 328	17.80 ± 1.80

For stored honey, stored pollen, and colony population, the five repeated measurements obtained during each year were first averaged within each colony. Data therefore represent mean ± SD of three independent colonies per management system (*n* = 3). Total honey production represents annual production per colony and is likewise expressed as mean ± SD of three colonies.

Data are expressed as mean ± SD of three independent colonies per management system (n = 3). For hive-stored honey, hive-stored pollen, and colony population, repeated measurements were first averaged within each colony. Different superscript letters indicate a significant difference between management systems within the same year (Welch’s two-sample t-test, p < 0.05). Superscript letters are shown only for statistically significant comparisons.

Bold values indicate a statistically significant difference between management systems within the same year (Welch’s two-sample t-test, p < 0.05).

Annual honey production was significantly higher in migratory than in stationary colonies in 2022 (20.83 ± 3.04 vs. 12.20 ± 2.14 kg; *p* = 0.0195). No significant differences between management systems were detected for hive-stored honey, hive-stored pollen, or colony population during the same year (*p* > 0.05). Thus, although migratory colonies showed numerically higher mean values for hive-stored honey and colony population in 2022, these differences were not statistically supported.

In 2023, none of the investigated colony-performance variables differed significantly between management systems (*p* > 0.05). Migratory colonies nevertheless showed numerically higher mean values for hive-stored pollen (1.34 ± 0.27 vs. 1.00 ± 0.13 kg), colony population (20,700 ± 328 vs. 19,150 ± 1,489 adult bees), and annual honey production (17.80 ± 1.80 vs. 14.47 ± 5.04 kg), whereas hive-stored honey was essentially comparable between management systems (3.19 ± 0.41 vs. 3.20 ± 0.68 kg). Given the limited number of independent colonies per management system, these numerical differences should be interpreted cautiously and should not be considered evidence of a management effect in the absence of statistical significance.

Overall, the clearest difference in colony performance was observed for annual honey production in 2022, when migratory colonies produced significantly more honey than stationary colonies. However, this difference was not reproduced in 2023, and no consistent significant effect of management was detected for the other colony-performance parameters. These findings therefore indicate that the productive response varied between years and among the parameters investigated. Differences in floral resource availability and other environmental conditions associated with the two management systems may have contributed to the observed patterns; however, the present experimental design does not allow the effects of individual environmental factors to be separated.

The colony-performance results should also be interpreted independently from the observed variation in gut bacterial community composition. The present analyses do not establish a causal relationship between microbiota variation and colony productivity. Similarly, the persistence of dominant core bacterial taxa across sampling periods indicates compositional continuity of major members of the honey bee gut microbiota, but does not by itself demonstrate functional stability, enhanced resilience, or the absence of dysbiosis. Functional properties of the bacterial communities were not directly measured in the present study.

Seasonal variation in bacterial community composition may reflect changes in diet, floral resources, environmental exposure, or other seasonally varying factors. Several dominant honey bee-associated bacterial taxa, including *Gilliamella*, *Snodgrassella*, and *Bartonella*, have previously been associated with important metabolic and host-related functions (20, 78, 87). Nevertheless, their persistence or variation in relative abundance in the present study should not be interpreted as direct evidence of changes in microbial metabolic activity, ecological adaptation, or improved colony performance.

Taken together, the colony-performance data indicate a significantly higher honey production in migratory colonies in 2022, whereas no significant differences in the investigated performance parameters were detected between management systems in 2023. The observed temporal variation in gut bacterial communities occurred alongside these differences in colony performance, but the present study does not establish a functional or causal connection between the two. The higher honey production recorded for migratory colonies in 2022 therefore indicates a productive advantage under the environmental conditions prevailing during that year rather than a consistent effect of migratory management across years. Further studies involving a larger number of independent colonies and direct measurements of microbial functional activity would be required to determine whether, and to what extent, gut microbiota dynamics contribute to colony performance under migratory beekeeping.

## 4 Conclusion

The present two-year study shows that the gut bacterial communities of Apis mellifera ligustica undergo marked temporal variation under both stationary and short-distance migratory beekeeping management. Dominant honey bee-associated taxa, particularly Gilliamella and Snodgrassella, persisted across seasons and years, although their relative abundances varied considerably over time and between management conditions. These findings indicate compositional continuity of major gut bacterial members alongside substantial temporal variation in community structure.

Colony-performance responses were not consistent across years. Migratory colonies showed significantly higher annual honey production than stationary colonies in 2022, whereas no significant differences in the investigated performance parameters were detected between management systems in 2023. Thus, the productive advantage observed in 2022 should be interpreted in the context of the environmental conditions prevailing during that year rather than as evidence of a general positive effect of migratory management on colony productivity.

Although temporal variation in gut bacterial communities occurred alongside differences in colony performance, the present study does not establish a causal or functional relationship between these responses. In particular, the persistence of dominant bacterial taxa should not be interpreted as evidence of functional stability, absence of dysbiosis, or enhanced microbiome resilience, as microbial functions were not directly assessed.

Overall, these findings contribute to understanding how honey bee gut bacterial communities vary over time under contrasting beekeeping management conditions and highlight the importance of considering both seasonal and interannual variability when evaluating the effects of short-distance migratory beekeeping. Future studies involving a larger number of independent colonies and apiaries, together with direct functional characterization of the microbiome and detailed assessment of dietary and environmental variables, will be required to clarify the mechanisms linking management practices, gut microbiota dynamics, and colony performance.

## Data Availability

The original contributions presented in the study are publicly available. This data can be found here: Mendeley Data, doi: 10.17632/fm78kwmdry.2.

## References

[1] Steinhauer N Kulhanek K Antúnez K Human H Chantawannakul P Chauzat M-P . Drivers of colony losses. Curr Opin Insect Sci. (2018) 26:142–8. doi: 10.1016/j.cois.2018.02.004 29764654

[2] Hristov P Shumkova R Palova N Neov B . Factors associated with honey bee colony losses: A mini-review. Veterinary Sci. (2020) 7:166. doi: 10.3390/vetsci7040166 33143134 PMC7712510

[3] Rowland BW Rushton SP Shirley MDF Brown MA Budge GE . Identifying the climatic drivers of honey bee disease in England and Wales. Sci Rep. (2021) 11:21953. doi: 10.1038/s41598-021-01495-w 34754028 PMC8578631

[4] Osterman J Aizen MA Biesmeijer JC Bosch J Howlett BG Inouye DW . Global trends in the number and diversity of managed pollinator species. Agric Ecosyst Environ. (2021) 322:107653. doi: 10.1016/j.agee.2021.10765342574925

[5] Frizzera D Zanni V D’Agaro M Boaro G Andreuzza L Del Fabbro S . Varroa destructor exacerbates the negative effect of cold contributing to honey bee mortality. J Insect Physiol. (2023) 151:104571. doi: 10.1016/j.jinsphys.2023.104571 37832840

[6] Motta EVS Moran NA . The honeybee microbiota and its impact on health and disease. Nat Rev Microbiol. (2024) 22:122–37. doi: 10.1038/s41579-023-00990-3 38049554 PMC10998682

[7] Kwong WK Moran NA . Gut microbial communities of social bees. Nat Rev Microbiol. (2016) 14:374–84. doi: 10.1038/nrmicro.2016.43 27140688 PMC5648345

[8] Meehan DE O’Toole PW . A review of diet and foraged pollen interactions with the honeybee gut microbiome. Microb Ecol. (2025) 88:54. doi: 10.1007/s00248-025-02551-y 40423805 PMC12116653

[9] Kwong WK Medina LA Koch H Sing K-W Soh EJY Ascher JS . Dynamic microbiome evolution in social bees. Sci Adv. (2017) 3:e1600513. doi: 10.1126/sciadv.1600513 28435856 PMC5371421

[10] Rothman JA Carroll MJ Meikle WG Anderson KE McFrederick QS . Longitudinal effects of supplemental forage on the honey bee (Apis mellifera) microbiota and inter- and intra-colony variability. Microb Ecol. (2018) 76:814–24. doi: 10.1007/s00248-018-1151-y 29397399

[11] Khan KA Al-Ghamdi AA Ghramh HA Ansari MJ Ali H Alamri SA . Structural diversity and functional variability of gut microbial communities associated with honey bees. Microb Pathogen. (2020) 138:103793. doi: 10.1016/j.micpath.2019.103793 31626917

[12] Powell JE Martinson VG Urban-Mead K Moran NA . Routes of acquisition of the gut microbiota of the honey bee Apis mellifera. Appl Environ Microbiol. (2014) 80:7378–87. doi: 10.1128/AEM.01861-14 25239900 PMC4249178

[13] Dong Z-X Li H-Y Chen Y-F Wang F Deng X-Y Lin L-B . Colonization of the gut microbiota of honey bee (Apis mellifera) workers at different developmental stages. Microbiol Res. (2020) 231:126370. doi: 10.1016/j.micres.2019.126370 31739261

[14] Bleau N Bouslama S Giovenazzo P Derome N . Dynamics of the honeybee (Apis mellifera) gut microbiota throughout the overwintering period in Canada. Microorganisms. (2020) 8:1146. doi: 10.3390/microorganisms8081146 32751209 PMC7464175

[15] Miller DL Smith EA Newton ILG . A bacterial symbiont protects honey bees from fungal disease. mBio. (2021) 12:10.1128/mbio.00503–21. doi: 10.1128/mbio.00503-21 34101488 PMC8262860

[16] Steele MI Motta EVS Gattu T Martinez D Moran NA . The gut microbiota protects bees from invasion by a bacterial pathogen. Microbiol Spectr. (2021) 9:e00394-21. doi: 10.1128/Spectrum.00394-21 34523998 PMC8557934

[17] Smutin D Lebedev E Selitskiy M Panyushev N Adonin L . Micro"bee"ota: Honey bee normal microbiota as a part of superorganism. Microorganisms. (2022) 10:2359. doi: 10.3390/microorganisms10122359 36557612 PMC9785237

[18] Gouda MNR Subramanian S Kumar A Ramakrishnan B . Microbial ensemble in the hives: deciphering the intricate gut ecosystem of hive and forager bees of Apis mellifera. Mol Biol Rep. (2024) 51:262. doi: 10.1007/s11033-024-09239-5 38302671

[19] Anderson KE Ricigliano VA . Honey bee gut dysbiosis: a novel context of disease ecology. Curr Opin Insect Sci. (2017) 22:125–32. doi: 10.1016/j.cois.2017.05.020 28805634

[20] Horak RD Leonard SP Moran NA . Symbionts shape host innate immunity in honeybees. Proc R Soc B Biol Sci. (2020) 287:20201184. doi: 10.1098/rspb.2020.1184 32842927 PMC7482289

[21] Maes PW Rodrigues PAP Oliver R Mott BM Anderson KE . Diet-related gut bacterial dysbiosis correlates with impaired development, increased mortality and Nosema disease in the honeybee (Apis mellifera). Mol Ecol. (2016) 25:5439–50. doi: 10.1111/mec.13862 27717118

[22] Kešnerová L Emery O Troilo M Liberti J Erkosar B Engel P . Gut microbiota structure differs between honeybees in winter and summer. ISME J. (2020) 14:801–14. doi: 10.1038/s41396-019-0568-8 31836840 PMC7031341

[23] Paris L Peghaire E Moné A Diogon M Debroas D Delbac F . Honeybee gut microbiota dysbiosis in pesticide/parasite co-exposures is mainly induced by Nosema ceranae. J Invertebr Pathol. (2020) 172:107348. doi: 10.1016/j.jip.2020.107348 32119953

[24] Castelli L Branchiccela B Romero H Zunino P Antúnez K . Seasonal dynamics of the honey bee gut microbiota in colonies under subtropical climate. Microb Ecol. (2022) 83:492–500. doi: 10.1007/s00248-021-01756-1 33973059

[25] Li Y Leonard SP Powell JE Moran NA . Species divergence in gut-restricted bacteria of social bees. Proc Natl Acad Sci. (2022) 119:e2115013119. doi: 10.1073/pnas.2115013119 35467987 PMC9170019

[26] Lazarova S Lozanova L Neov B Shumkova R Balkanska R Palova N . Composition and diversity of bacterial communities associated with honey bee foragers from two contrasting environments. Bull Entomol Res. (2023) 113:693–702. doi: 10.1017/S0007485323000378 37545319

[27] Luo Z Xiong J Jiang M Li L Wang G Huang H . Co-treatment of swine manure and lignocellulosic biomass by liquefaction: Parameter optimization, product characterization, reaction mechanism. J Supercrit Fluids. (2024) 205:106138. doi: 10.1016/j.supflu.2023.10613842574925

[28] Kim H Maigoro AY Lee J-H Frunze O Kwon H-W . The improving effects of probiotic-added pollen substitute diets on the gut microbiota and individual health of honey bee (Apis mellifera L.). Microorganisms. (2024) 12:1567. doi: 10.3390/microorganisms12081567 39203409 PMC11356693

[29] Zumkhawala-Cook A Gallagher P Raymann K . Diet affects reproductive development and microbiota composition in honey bees. Anim Microbiome. (2024) 6:64. doi: 10.1186/s42523-024-00350-3 39501371 PMC11539837

[30] Brar G Ngor L McFrederick QS Torson AS Rajamohan A Rinehart J . High abundance of lactobacilli in the gut microbiome of honey bees during winter. Sci Rep. (2025) 15:7409. doi: 10.1038/s41598-025-90763-0 40032901 PMC11876673

[31] De Iorio MG Minozzi G Tiezzi F . Variation of gut microbiota composition in a honey bee breeding population: exploring potential links with docility and honey production. animal. (2025) 19:101474. doi: 10.1016/j.animal.2025.101474 40132528

[32] Braglia C Rudelli C Tinti A Bocquet M Isani G Bulet P . Unravelling pollen diet and microbiome influence on honey bee health. Sci Rep. (2025) 15:13474. doi: 10.1038/s41598-025-96649-5 40251206 PMC12008280

[33] Pilati L Prestamburgo M . Sequential relationship between profitability and sustainability: The case of migratory beekeeping. Sustainability. (2016) 8:94. doi: 10.3390/su801009430654563

[34] Wine MSE . Pollinators are essential workers. Dela J Public Health. (2021) 7:58–60. doi: 10.32481/djph.2021.01.010 34467180 PMC8352550

[35] Chakrabarti P Sagili RR . Chapter 8 - Managed foraging for honey and crop pollination—Honey bees as livestock The Foraging Behavior of the Honey Bee (Apis Mellifera, L) (2024). [place unknown]: Academic Press (Accessed 2025 Sept 19).

[36] Jara L Ruiz C Martín-Hernández R Muñoz I Higes M Serrano J . The effect of migratory beekeeping on the infestation rate of parasites in honey bee (Apis mellifera) colonies and on their genetic variability. Microorganisms. (2021) 9:22. doi: 10.3390/microorganisms9010022 33374609 PMC7822443

[37] Pocol CB Šedík P Brumă IS Amuza A Chirsanova A . Organic beekeeping practices in Romania: Status and perspectives towards a sustainable development. Agriculture. (2021) 11:281. doi: 10.3390/agriculture1104028130654563

[38] Feketéné Ferenczi A Szűcs I Bauerné Gáthy A . Evaluation of the pollination ecosystem service of the honey bee (Apis mellifera) based on a beekeeping model in Hungary. Sustainability. (2023) 15:9906. doi: 10.3390/su1513990630654563

[39] Kishan Tej M Aruna R Mishra G Srinivasan MR . Beekeeping in India Industrial Entomology (2017). Singapore: Springer (Accessed 2025 Sept 19).

[40] Zheng H-Q Cao L-F Huang S Neumann P Hu F . Current status of the beekeeping industry in China. In: Asian Beekeeping in the 21st Century (2018). Singapore: Springer Singapore. p. 129–58. doi: 10.1007/978-981-10-8222-1_6

[41] Albayrak A Çeven S Bayır R . Modeling of migratory beekeeper behaviors with machine learning approach using meteorological and environmental variables: The case of Turkey. Ecol Inf. (2021) 66:101470. doi: 10.1016/j.ecoinf.2021.10147042574925

[42] Lee KY Park J Lee SJ Kwak K-W Le YB Sankar K . Migratory beekeeping information system: Application to the study of distribution and movement patterns of migratory beehives in South Korea during the honey flow period. J Apiculture. (2023) 38:325–34. doi: 10.17519/apiculture.2023.11.38.4.325

[43] Vaudo AD Orr MC Zhou Q-S Zhu C-D Mu J López-Uribe MM . Low-density migratory beekeeping induces intermediate disturbance effects on native bee communities in Tibetan Plateau alpine meadows. J Insect Sci. (2024) 24:4. doi: 10.1093/jisesa/ieae108 39563069 PMC11576356

[44] Pavlin A Marinč A Prešern J . Go with the flow: a case study of migratory beekeeping and its associated costs. J Econ Entomol. (2025) 118:1485–94. doi: 10.1093/jee/toaf119 40568750 PMC12412298

[45] Simone-Finstrom M Li-Byarlay H Huang MH Strand MK Rueppell O Tarpy DR . Migratory management and environmental conditions affect lifespan and oxidative stress in honey bees. Sci Rep. (2016) 6:32023. doi: 10.1038/srep32023 27554200 PMC4995521

[46] Melicher D Wilson E Bowsher J Peterson S Yocum G Rinehart J . Long-distance transportation causes temperature stress in the honey bee, Apis mellifera (Hymenoptera: Apidae). Environ Entomol. (2019) 48:691–701. doi: 10.1093/ee/nvz027 30927358 PMC6554651

[47] Welch A Drummond F Tewari S Averill A Burand JP . Presence and prevalence of viruses in local and migratory honeybees (Apis mellifera) in Massachusetts. Appl Environ Microbiol. (2009) 75:7862–5. doi: 10.1128/AEM.01319-09 19854916 PMC2794090

[48] Zhu X Zhou S Huang ZY . Transportation and pollination service increase abundance and prevalence of Nosema ceranae in honey bees (Apis mellifera). J Apic Res. (2014) 53:469–71. doi: 10.3896/IBRA.1.53.4.06

[49] Giacobino A Pacini A Molineri A Bulacio Cagnolo N Merke J Orellano E . Environment or beekeeping management: What explains better the prevalence of honey bee colonies with high levels of *Varroa destructor*? Res Vet Sci. (2017) 112:1–6. doi: 10.1016/j.rvsc.2017.01.001 28095337

[50] Alger SA Burnham PA Lamas ZS Brody AK Richardson LL . Home sick: impacts of migratory beekeeping on honey bee (Apis mellifera) pests, pathogens, and colony size. PeerJ. (2018) 6:e5812. doi: 10.7717/peerj.5812 30405967 PMC6216951

[51] Jara L Lopez Martinez D Muñoz I Rua P . Epidemiological survey of Ascosphaera apis in small-scale migratory Apis mellifera iberiensis colonies. Sociobiology. (2018) 65:285. doi: 10.13102/sociobiology.v65i2.2685

[52] Martínez-López V Ruiz C De la Rúa P . Migratory beekeeping and its influence on the prevalence and dispersal of pathogens to managed and wild bees. Int J For Parasitology: Parasites Wildlife. (2022) 18:184–93. doi: 10.1016/j.ijppaw.2022.05.004 35663725 PMC9160285

[53] Cestaro LG Alves MLTMF Message D Silva MVGB Teixeira ÉW . Honey bee (Apis mellifera) health in stationary and migratory apiaries. Sociobiology. (2017) 64:42–53. doi: 10.13102/sociobiology.v64i1.1183

[54] Nishanthini K Kanagarajan R . The effect of migratory beekeeping on Indian honey bee (Apis cerana indica) microbiota – a first report. J Apic Res. (2025) 64:37–52. doi: 10.1080/00218839.2023.226459837339054

[55] Soares KO Hale VL Medeiros G Silva N Lima MV Rocha T . The honey bee gut microbiota: a review under the perspective of bees threatened by agrochemicals. OBSERVATÓRIO LA ECONOMÍA LATINOAMERICANA. (2023) 21:27315–36. doi: 10.55905/oelv21n12-207

[56] Zhang C Pokhrel S Wu Z Miao X Huang ZY Yang W . Longevity, food consumption, and foraging performance of Apis cerana and Apis mellifera in mixed colonies. Apidologie. (2019) 50:153–62. doi: 10.1007/s13592-018-0626-730311153

[57] Li X Ma W Shen J Long D Feng Y Su W . Tolerance and response of two honeybee species Apis cerana and Apis mellifera to high temperature and relative humidity. PloS One. (2019) 14:e0217921. doi: 10.1371/journal.pone.0217921 31170259 PMC6553758

[58] Dall’Olio R Marino A Lodesani M Moritz RFA . Genetic characterization of Italian honeybees, *Apis mellifera ligustica* , based on microsatellite DNA polymorphisms. Apidologie. (2007) 38:207–17. doi: 10.1051/apido:2006073

[59] Carroll MJ Brown N Goodall C Downs AM Sheenan TH Anderson KE . Honey bees preferentially consume freshly-stored pollen. PloS One. (2017) 12:e0175933. doi: 10.1371/journal.pone.0175933 28430801 PMC5400263

[60] Taurisano V Ribani A Sami D Nelson Johnson KE Schiavo G Utzeri VJ . Distribution of honey bee mitochondrial DNA haplotypes in an Italian region where a legislative act is protecting the Apis mellifera ligustica subspecies. Sci Rep. (2024) 14:20583. doi: 10.1038/s41598-024-71233-5 39232026 PMC11375103

[61] Iorizzo M Pannella G Succi M Ganassi S Di Criscio D Tedino C . Seasonal dynamics of the gut microbiota in Apis mellifera ligustica: a two-year longitudinal study. Front Insect Sci. (2026) 6:1920906. doi: 10.3389/finsc.2026.1920906 42601898 PMC13472841

[62] Iorizzo M Albanese G Letizia F Testa B Di Criscio D Petrarca S . Diversity of plant pollen sources, microbial communities, and phenolic compounds present in bee pollen and bee bread. Environ Sci pollut Res. (2025) 32:10425–35. doi: 10.1007/s11356-024-34517-x 39073714

[63] Cozzolino R Albanese G Matarazzo C Nigris A Ambrosone L De Cristofaro A . Chemical fingerprint of phenolamides and flavonoid glycosides in bee pollen and bee bread from different altitudes and botanical origins. Food Biosci. (2026) 80:108948. doi: 10.1016/j.fbio.2026.10894842574925

[64] Iorizzo M Ganassi S Albanese G Letizia F Testa B Tedino C . Antimicrobial activity from putative probiotic lactic acid bacteria for the biological control of American and European foulbrood diseases. Veterinary Sci. (2022) 9:236. doi: 10.3390/vetsci9050236 35622764 PMC9143654

[65] Walker AW Martin JC Scott P Parkhill J Flint HJ Scott KP . 16S rRNA gene-based profiling of the human infant gut microbiota is strongly influenced by sample processing and PCR primer choice. Microbiome. (2015) 3:26. doi: 10.1186/s40168-015-0087-4 26120470 PMC4482049

[66] Abellan-Schneyder I Matchado MS Reitmeier S Sommer A Sewald Z Baumbach J . Primer, pipelines, parameters: Issues in 16S rRNA gene sequencing. mSphere. (2021) 6:e01202-20. doi: 10.1128/mSphere.01202-20 33627512 PMC8544895

[67] Caporaso JG Kuczynski J Stombaugh J Bittinger K Bushman FD Costello EK . QIIME allows analysis of high-throughput community sequencing data. Nat Methods. (2010) 7:335–6. doi: 10.1038/nmeth.f.303 20383131 PMC3156573

[68] Edgar RC Haas BJ Clemente JC Quince C Knight R . UCHIME improves sensitivity and speed of chimera detection. Bioinformatics. (2011) 27:2194–200. doi: 10.1093/bioinformatics/btr381 21700674 PMC3150044

[69] Rognes T Flouri T Nichols B Quince C Mahé F . VSEARCH: a versatile open source tool for metagenomics. PeerJ. (2016) 4:e2584. doi: 10.7717/peerj.2584 27781170 PMC5075697

[70] Eren AM Morrison HG Lescault PJ Reveillaud J Vineis JH Sogin ML . Minimum entropy decomposition: Unsupervised oligotyping for sensitive partitioning of high-throughput marker gene sequences. ISME J. (2015) 9:968–79. doi: 10.1038/ismej.2014.195 25325381 PMC4817710

[71] Angly FE Dennis PG Skarshewski A Vanwonterghem I Hugenholtz P Tyson GW . CopyRighter: a rapid tool for improving the accuracy of microbial community profiles through lineage-specific gene copy number correction. Microbiome. (2014) 2:11. doi: 10.1186/2049-2618-2-11 24708850 PMC4021573

[72] Imdorf A Gerig L . Course in determination of colony strength. Schweiz Bienen-Zeitung. (2001) 106.

[73] Delaplane KS van der Steen J Guzman-Novoa E . Standard methods for estimating strength parameters of Apis mellifera colonies. J Apic Res. (2013) 52:1–12. doi: 10.3896/IBRA.1.52.1.03

[74] Dietemann V Neumann P Carreck N Ellis JD . The COLOSS BEEBOOK – Volume III, Part 1: Standard methods for Apis mellifera product research. J Apic Res. (2019) 58:1–2. doi: 10.1080/00218839.2019.157444937339054

[75] Dainat B Dietemann V Imdorf A Charrière J-D . A scientific note on the ‘Liebefeld Method’ to estimate honey bee colony strength: its history, use, and translation. Apidologie. (2020) 51:422–7. doi: 10.1007/s13592-019-00728-230311153

[76] Guzman-Novoa E Morfin N Dainat B Williams GR van der Steen J Correa-Benítez A . Standard methods to estimate strength parameters, flight activity, comb construction, and fitness of Apis mellifera colonies 2.0. J Apic Res. (2025) 64:533–54. doi: 10.1080/00218839.2024.232985337339054

[77] Hernandez J Maisonnasse A Cousin M Beri C Le Quintrec C Bouetard A . ColEval: honeybee COLony structure EVALuation for field surveys. Insects. (2020) 11:41. doi: 10.3390/insects11010041 31948048 PMC7023294

[78] Kešnerová L Mars RAT Ellegaard KM Troilo M Sauer U Engel P . Disentangling metabolic functions of bacteria in the honey bee gut. PloS Biol. (2017) 15:e2003467. doi: 10.1371/journal.pbio.2003467 29232373 PMC5726620

[79] Bonilla-Rosso G Engel P . (2018). Functional roles and metabolic niches in the honey bee gut microbiota. Curr Opinion Microbiol. 43:69–76. doi: 10.1016/j.mib.2017.12.009 29309997

[80] D’Alvise P Hoffmann V Gihring K Kieboom M Hasselmann M . Seasonal dynamics and co‐occurrence patterns of honey bee pathogens revealed by high‐throughput RT‐qPCR analysis. Ecol Evol. (2019) 9:10241–52. doi: 10.1002/ece3.5544 31624548 PMC6787843

[81] Alaux C Ducloz F Crauser D Le Conte Y . Diet effects on honeybee immunocompetence. Biol Lett. (2010) 6:562–5. doi: 10.1098/rsbl.2009.0986 20089536 PMC2936196

[82] Raymann K Moran NA . The role of the gut microbiome in health and disease of adult honey bee workers. Curr Opin Insect Sci. (2018) 26:97–104. doi: 10.1016/j.cois.2018.02.012 29764668 PMC6010230

[83] Douglas AE . Fundamentals of Microbiome Science: How Microbes Shape Animal Biology. Princeton, NJ: Princeton University Press (2018).

[84] Taha E-K AL-Kahtani S . Relationship between population size and productivity of honey bee colonies. J Entomology. (2013) 10:163–9. doi: 10.3923/je.2013.163.169

[85] Anderson KE Carroll MJ Sheehan T Mott BM Maes P Corby-Harris V . Hive-stored pollen of honey bees: many lines of evidence are consistent with pollen preservation, not nutrient conversion. Mol Ecol. (2014) 23:5904–17. doi: 10.1111/mec.12966 25319366 PMC4285803

[86] Fernandes KE Frost EA Remnant EJ Schell KR Cokcetin NN Carter DA . The role of honey in the ecology of the hive: Nutrition, detoxification, longevity, and protection against hive pathogens (2022) (Accessed January 19, 2026). 10.3389/fnut.2022.954170 PMC9359632 35958247

[87] Zheng H Perreau J Powell JE Han B Zhang Z Kwong WK . Division of labor in honey bee gut microbiota for plant polysaccharide digestion. Proc Natl Acad Sci. (2019) 116:25909–16. doi: 10.1073/pnas.1916224116 31776248 PMC6926048

