# Supplementary material for: Seasonal gut microbiota dynamics and colony performance under stationary and short-distance migratory beekeeping in Apis mellifera ligustica
Source: Front Insect Sci. 2026 Oct 8;6:1890935. doi: 10.3389/finsc.2026.1890935 (PMC13687282; doi:10.3389/finsc.2026.1890935)
Supplement: Supplementary file 1 [file DataSheet1.zip › Supplementary material/ Supplementary figures CAPTION.docx]

**Figure S1.** Class-level composition of honey bee gut bacterial communities in 2022 and 2023. Stacked bars represent the mean relative abundance (%) of the principal bacterial classes across the three independent colonies (n = 3) for each season under stationary (A) and migratory (B) beekeeping management. Taxa not individually displayed were grouped as “Other taxa”.

**Figure S2.** Order-level composition of honey bee gut bacterial communities in 2022 and 2023. Stacked bars represent the mean relative abundance (%) of the principal bacterial orders across the three independent colonies (n = 3) for each season under stationary (A) and migratory (B) beekeeping management. Taxa not individually displayed were grouped as “Other taxa”.

**Figure S3.** Family-level composition of honey bee gut bacterial communities in 2022 and 2023. Stacked bars represent the mean relative abundance (%) of the principal bacterial families across the three independent colonies (n = 3) for each season under stationary (A) and migratory (B) beekeeping management. Taxa not individually displayed were grouped as “Other taxa”.

**Figure S4.** Species-level composition of honey bee gut bacterial communities in 2022 and 2023. Stacked bars represent the mean relative abundance (%) of the principal bacterial taxa assigned at the species level across the three independent colonies (n = 3) for each season under stationary (A) and migratory (B) beekeeping management. Species-level assignments derived from partial V1–V3 16S rRNA gene sequences should be interpreted cautiously and are presented for descriptive purposes only.
